# A Case Study of the Response of Immunogenic Gluten Peptides to Sourdough Proteolysis

**DOI:** 10.3390/nu13061906

**Published:** 2021-06-01

**Authors:** Olivia J. Ogilvie, Juliet A. Gerrard, Sarah Roberts, Kevin H. Sutton, Nigel Larsen, Laura J. Domigan

**Affiliations:** 1 School of Biological Sciences, University of Canterbury, 20 Kirkwood Avenue, Upper Riccarton, Christchurch 8041, New Zealand; 2 Riddet Institute, Massey University, Private Bag 11 222, Palmerston North 4442, New Zealand; j.gerrard@auckland.ac.nz (J.A.G.); Kevin.Sutton@plantandfood.co.nz (K.H.S.); l.domigan@auckland.ac.nz (L.J.D.); 3 School of Biological Sciences, University of Auckland, Private Bag 92019, Auckland 1142, New Zealand; 4 The New Zealand Institute for Plant & Food Research Limited, Private Bag 4704, Christchurch Mail Centre, Christchurch 8140, New Zealand; sarah.roberts@plantandfood.co.nz (S.R.); nigellarsen1@gmail.com (N.L.); 5 Department of Chemical and Materials Engineering, University of Auckland, Private Bag 92019, Auckland 1142, New Zealand

**Keywords:** sourdough, fermentation, gluten, antigen, immunogenic peptide, mass spectrometry

## Abstract

Celiac disease is activated by digestion-resistant gluten peptides that contain immunogenic epitopes. Sourdough fermentation is a potential strategy to reduce the concentration of these peptides within food. However, we currently know little about the effect of partial sourdough fermentation on immunogenic gluten. This study examined the effect of a single sourdough culture (representative of those that the public may consume) on the digestion of immunogenic gluten peptides. Sourdough bread was digested via the INFOGEST protocol. Throughout digestion, quantitative and discovery mass spectrometry were used to model the kinetic release profile of key immunogenic peptides and profile novel peptides, while ELISA probed the gluten’s allergenicity. Macrostructural studies were also undertaken. Sourdough fermentation altered the protein structure, in vitro digestibility, and immunogenic peptide release profile. Interestingly, sourdough fermentation did not decrease the total immunogenic peptide concentration but altered the in vitro digestion profile of select immunogenic peptides. This work demonstrates that partial sourdough fermentation can alter immunogenic gluten digestion, and is the first study to examine the in vitro kinetic profile of immunogenic gluten peptides from sourdough bread.

## 1. Introduction

Celiac disease is a systemic CD4+ T-cell mediated autoimmune disease, activated by discrete epitopes within the primary sequence of gluten proteins [1]. Celiac epitopes are nine amino acids in length and generally reside within stretches of amino acids rich in proline, rendering both the epitopes and adjacent residues resistant to gastrointestinal proteolysis [2,3,4]. Because of their proteolytic resistance, peptide fragments of gluten persist throughout the gastrointestinal digestion of gluten-containing foods [4,5]. Notably, not all digestion-resistant gluten peptide fragments harbour celiac epitopes. When epitopes are present within a peptide, they are deemed an immunogenic peptide [6,7,8]. Hundreds of unique immunogenic gluten peptides have been identified during the digestion of wheat gluten proteins, ranging between 10 and 33 amino acids in length [4,9]. These peptides are heterogeneous in amino acid composition and exhibit a hierarchy of immunodominance, dictated by both the epitope itself and residues flanking the epitope [10]. The ‘33mer peptide’ is a 33 amino acid immunogenic peptide derived from the protein alpha2-gliadin that contains six overlapping epitopes; it is immunodominant and the most widely discussed immunogenic peptide within the literature [4,11]. However, many other immunogenic peptides play a critical role in the pathogenesis of celiac disease [2,4,12]. 

Gluten proteins fall into five classes, dependent on their amino acid composition and consequent solubility. All gluten protein classes contain celiac epitopes and therefore produce immunogenic gluten peptides during digestion [13]. As summarised by Wang et al. [14], the α/β-gliadins and γ-gliadins are monomeric, with an amino acid composition of 2–3% cysteine and 15–20% proline residues. The ω-gliadins are also monomeric, possessing no cysteines and 20–30% proline residues. The high-molecular-weight glutenin proteins are polymeric within an amino acid composition of 15–20% proline and 0.5–1.5% cysteine. Similarly, the low-molecular-weight glutenins are polymeric, with 2–3% cysteine and 30–45% proline. All gliadin proteins are alcohol-soluble, while the glutenins are alcohol insoluble [14]. Generally, the gliadin class exhibits higher protease resistance than the glutenin class and more commonly harbours immunodominant epitopes and peptides [8].

The only current treatment for celiac disease is to remove immunogenic gluten peptides from the diet via a strict and life-long gluten-free diet. Numerous alternative strategies are being explored that aim to decrease the concentration of immunogenic peptides within food products. These range from biotechnology-based methods such as plant engineering and plant breeding [15] to food processing methods such as sourdough fermentation [16]. Each strategy varies in its efficiency to create products that comply with a gluten-free diet.

Compared to modern fast fermentation bread leavened with baker’s yeast (*Saccharomyces cerevisiae*), sourdough bread is leavened with a starter culture containing flour, water, lactic acid bacteria (LAB), and various yeasts. During sourdough fermentation, gluten proteins undergo proteolysis. If proteolysis occurs within a region of gluten that contains an epitope, the concentration of immunogenic gluten peptides within wheat bread consequently decreases as the epitope is destroyed [17,18,19,20]. Gluten protein proteolysis occurs via one of two pathways. The dominant process is primary proteolysis, mediated by endogenous flour enzymes’ activation due to dough acidification [18,21]. These enzymes optimally function at pH 3.5-5.0 and include cysteine proteases, serine carboxypeptidase II, and aspartic proteases [22].

Secondary proteolysis occurs when proteases are secreted from the LAB and yeasts within the active sourdough culture [23]. The precise microbial composition of sourdough is variable, primarily dictated by the feeding flour’s endogenous microbial composition [20]. Some strains of sourdough microflora secrete enzymes, such as prolyl endopeptidases, that can efficiently degrade proline-X bonds. These sourdough cultures exhibit an enhanced ability to degrade gluten proteins and immunogenic gluten peptides [18]. Specific combinations of the microbes with enhanced ability to degrade gluten can produce bread with lower reactive gluten contents [19] and reduced celiac immunogenicity [24]. The digestion kinetics and gluten degradation mechanism within these ferments are well-characterised throughout the literature [24].

The cultures used in sourdoughs consumed by the public are somewhat different to those with enhanced degradation ability, as they are selected for both their sensory profile and baking performance effects. Their ability to degrade gluten proteins is reduced when compared to optimised cultures, resulting in some gluten proteins remaining intact following sourdough fermentation. There is a lack of understanding surrounding the effect of partial sourdough fermentation on the profile of immunogenic gluten peptides produced during the digestion of sourdough bread. 

This work undertook a case study of a single sourdough bread representative of those consumed by the public and compared its immunogenic peptide profile with a modern fast fermentation bread. The degree of gluten proteolysis and alterations in gluten protein structure were investigated using high-pressure liquid chromatography (HPLC) and confocal microscopy, respectively. The breads were digested using an in vitro simulated human digestion, the resulting immunogenic peptide profile and total antigenicity was then investigated using targeted plus untargeted mass spectrometry (MS) and enzyme-link immunosorbent assay (ELISA), respectively. This is the first study to model the effect of partial sourdough fermentation on the immunogenic peptide profile within a bread, contributing consumer-relevant knowledge to sourdough and celiac disease proteomics and producing a workflow for further research.

## 2. Materials and Methods

### 2.1. Materials 

White flour was purchased from Champion Flour Milling Ltd. (Champion Epic Bakers Flour (Product Code: 56472) protein content 12%, ash content 0.55%, and falling number 300 s). High-purity water was produced using a Milli-Q^®^ Advantage A10 Water Purification System. The NaCl used during baking was analytical quality purchased from Sigma Aldrich. Acetonitrile (ACN), trifluoracetic acid (TFA), and formic acid were purchased at LC–MS quality from Thermo Fisher Scientific (Rockford, IL, USA). Ethanol and methanol were AnalaR grade from Thermo Fisher Scientific (Rockford, IL, USA). Rhodamine B and fluorescein isothiocyanate (FITC) were purchased from Sigma-Aldrich. Seven synthetic peptides were purchased from New England Peptide (Gardner, MA, USA) at >98% purity. 

### 2.2. Bread Preparation

White fast fermentation bread (developed using mechanical dough development (MDD)) and sourdough bread were prepared as described below using equivalent protocols and reagents. Champion Epic Flour was used to prepare both breads. Yeast was replaced in sourdough bread with an artisan type 1 sourdough culture. Throughout bread preparation, dough samples were collected and preserved by snap-freezing in liquid nitrogen and storage at −20 °C. 

#### 2.2.1. White Fast Fermentation Bread Preparation

White bread was prepared as described previously [8] by mechanical dough development (MDD) using a doughLAB (Perten Instruments). Briefly, doughs were prepared to contain 300 g flour, 6.01 g salt, 2.26 g sugar, 0.03 g ascorbic acid, 3.6 g powdered bread emulsifier, and 9.41 g of yeast. Dry ingredients were combined for 1 min at 63 rpm, and then water was added based on a pre-determined water absorbance of 62%. Doughs were formed by mixing at 120 rpm until a work input (Wh.kg^−1^) of 12 was reached. Doughs were proved for 10 min at 30 ± 2 °C, moulded by one sheeting pass, then proved and baked for 45 min, 40 ± 2°C at 82% humidity, and 23 min at 218 °C (respectively). Breads were sliced, freeze-dried (VirTis Genesis Pilot Lyophilizer), then stored at −20 °C. Breads were prepared in duplicate.

#### 2.2.2. Sourdough Preparation

A type 1 sourdough was prepared using a sourdough starter raised on white flour. The starter was fed morning and night by the addition of flour and milliQ water at 50:50 *w*/*w*. Half of the existing culture by weight was removed and replaced with the new flour–water mix during feeding. The culture was deemed active when its volume doubled within two hours of feeding. When required, culture dormancy was induced for up to six months by incubation at 4 °C. 

The active culture was used to formulate sourdough. Each dough contained 154 g of active culture (41% *w*/*w*), 146 g of flour (39% *w*/*w*), 52 g of milliQ water (14% *w*/*w*), and 6.01 g of salt (6% *w*/*w*). Doughs were formulated and mixed in a doughLAB (Perten Instruments) with a bowl temperature of 30 °C for 5 min at 120 rpm. Following mixing, dough fermentation was undertaken for 12–16 h at 22 °C, 80% humidity. A loaf was then hand-shaped, fermented for an additional 4 h at the same conditions, and baked at 190 °C for 50 min. Breads were sliced, freeze-dried (VirTis Genesis Pilot Lyophilizer), then stored at −20 °C. Samples were prepared in duplicate. 

### 2.3. Confocal Microscopy 

Confocal microscopy was undertaken using a Leica TCS SP5 (Leica Microsystems) confocal Microscope equipped with the following lasers: 405 nm (violet), 514 nm (blue), 561 nm (green), and 633 nm (red) [25]. Data were collected using a 63× oil immersion lens (numerical aperture 0.7) and refraction index of 1.52. Samples were sliced with a razor blade 2 × 5 mm (approximately) then stained with solution containing 0.066% (*w*/*v*) rhodamine B (protein stain) and 0.002% (*w*/*v*) FITC (starch stain) in water. Approximately 50 μL of dye was added to each sample, which was then left for five minutes in the dark prior to imaging in a FluoroDish (35 mm diameter, Thermo Fisher Scientific (Rockford, IL, USA)). Images were collected in sequential scan mode using the 488 nm laser then the 561 nm laser. The scan speed was 400 Hz, scan mode xyz, pinhole 95.5 μm, pixel size 1024 × 1024, step size 1.09 μm, with six ‘line averages’. Three biological and three technical replicates were examined for each condition. The resulting images were visualised in ImageJ and LAS X software (Leica Microsystems). 

### 2.4. HPLC

Protein was extracted for HPLC using the modified Heubner & Bietz protocol [26,27] in duplicate. Briefly, freeze-dried bread was crushed and gliadin extracted by inversion for 30 min at 100 mg.mL^−1^ in 70% (*v*/*v*) ethanol. The sample was centrifuged at 10,000× *g*, supernatant collected, and extraction repeated but at 200 mg.mL^−1^. The subsequent supernatants were combined and analysed. 

HPLC quantification of gliadin proteins was undertaken as described previously [28] using a 150 × 4.6 mm Zorbax 300SB-C8 column fitted with a 12.5 × 4.6 mm guard column (same packing) using a Waters 2695 ‘Alliance’ solvent delivery control system, attached to a Waters 2478 ultraviolet–visible detector (Waters Corporation), controlled using Waters Empower software. Separation employed mobile phases A (99.9% water, 0.1% TFA) and B (99.9% ACN, 0.1% TFA). Mobile phase C contained 100% water and mobile phase D 100% ACN. The sample injection volume was 20 μL. Chromatographic separation was undertaken over 86 min. Changes in buffer composition occurred linearly at a flow rate of 0.5 mL.min^−1^ using the following schedule: 0–60 min B 20%; 60–70 min B 50%; 70–71 min B 20%; 71–86 min D 20%. Each run included bovine serum albumin as a standard. Absorbance data were collected at 210 nm, gliadin fractions were determined using typical retention patterns [28], and concentration was determined using linear regression of the protein standard. Statistical analyses of data were undertaken using Prism.

### 2.5. Mass Spectrometry 

All MS was undertaken in positive ion mode on an Orbitrap Q Exactive™ Plus (Thermo Fisher Scientific, Waltham, MA, USA) coupled to a Vanquish uHPLC System (Thermo Fisher Scientific, Waltham, MA, USA). The instrument was calibrated before each run using Pierce LTQ Velos ESI Positive Ion Calibration Solution (Thermo Fisher Scientific, Waltham, MA, USA). Chromatography was undertaken on a pre-equilibrated, reversed-phase Aeris 1.7 μm PEPTIDE XB-C18 100 Å, LC Column 150 × 2.1 mm (Phenomenex, Torrance, CA, USA) fitted with a Security Guard ULTRA cartridge, uHPLC C18 Peptide (Phenomenex, Torrance, CA, USA). Two mobile phases were used during chromatography: mobile phase A (99.9% milliQ water, 0.1% trifluoroacetic acid (TFA)), and mobile phase B (99.9% ACN 0.1% TFA).

#### 2.5.1. Sample Preparation

Before MS analysis, all samples were digested using the sequential three-step INFOGEST [29] static simulated in vitro digestion, as described previously [30]. As detailed in Mineskus et al. [29] ×1.25 stocks of simulated oral fluids (SOF), simulated gastric fluids (SGF), and simulated intestinal fluids (SIF) were prepared and stored at −20 °C. The enzymatic activities of porcine α-amylase, porcine pepsin, porcine chymotrypsin, and porcine trypsin were determined as recommended within Mineskus et al. [29]. The total digestion duration was 242 min and was undertaken at 37 °C in a shaking water bath. First, a 3 g sample of freeze-dried bread was rehydrated with 2 mL of milliQ water. Oral fluids were added to the sample at 50:50 *w*/*w* to a final concentration of ×1 SOF, 75 U.mL^−1^ α-amylase, 0.75 mmol.L^−1^ CaCl_2_ at pH 7, and incubated for two minutes. Gastric fluids were added sequentially at 50:50 *w*/*v* with the bread mass to a final concentration of ×1 SGF, 2000 U.mL^−1^ pepsin, 0.075 mmol.L^−1^ CaCl_2_, pH 3, for 120 min. Simulated intestinal fluids were then added at 50:50 *w*/*w* with the bread mass to a final concentration of ×1 SIF, 25 U.mL^−1^ α-chymotrypsin, 100 U.mL^−1^ trypsin, and 0.3 mmol.L^−1^ CaCl_2_, pH 7, for a final 120 min. At the desired time-points throughout digestion, aliquots were removed, and the reaction was quenched by the addition of 0.5% *v*/*v* trifluoroacetic acid, then snap-frozen and stored at −20 °C. Samples were digested in triplicate.

Digested samples were prepared for MS analysis by solid-phase extraction of the digesta supernatant, as described previously [30]. Briefly, digesta were defrosted, centrifuged at 10,000× *g* for 10 min, then the supernatant was collected. An isotopically labelled internal standard of P1, referred to as P1-heavy (P1H), was added to the supernatant at 3 μg.mL^−1^, then 200 μL of sample was loaded into the pre-prepared cartridge. Unbound molecules were removed by washing with 2 mL of 5% ACN, then target compounds eluted 2 × 210 μL aliquots of 80% ACN and 1% formic acid (FA). Eluate was then filtered using a 0.2 μm SINGLE StEP filter vial (Thomson Instrument Company), then analysed by MS within 24 h.

#### 2.5.2. Targeted LC-MS

Six immunogenic gluten peptides were monitored using label-free targeted mass spectrometry (Table 1), as described previously [30]. For each peptide, data were collected in parallel reaction monitoring (PRM) using the scheduled transitions described by Ogilvie et al. [30,31] and in Supplementary file 1. 

For sample analysis, 2 μL of digested sample was injected onto the column then eluted at 40 °C as follows: 0–4 min, B 7–40%, flow rate 0.3 mL.min^−1^; 4–12 min B 95%, flow rate 0.4 mL.min^−1^; 12–15 min B 7%, flow rate 0.3 mL.min^−1^. Data collection was undertaken using a spray voltage of 3.5 kV, spray current of 17 μA, aux gas flow rate of 10, sheath gas flow rate of 45, scan range *m*/*z* 108–4015, resolving power of 70,000, the capillary temperature of 320 °C, normalised collision energy of stepped 18–27, and mass error 0.05 Da. An external standard curve was constructed for all seven peptides at 0.5, 1, 2, 5, and 10 μg.mL^−1^ for each run.

Data were processed in the Xcalibur Quan Browser (Version 4.0, Thermo Fisher Scientific, Waltham, MA, USA). Peptides were identified by the *m*/*z* and quantifier fragment ions shown in Supplementary file 1. The total ion chromatogram was extracted to determine the peptide abundance. The peptide concentration was determined by linear regression of the standard curve in Xcalibur Quan. Statistical analyses were undertaken in Prism.

#### 2.5.3. Untargeted LC-MS

Untargeted MS analyses were undertaken in data-dependent analysis (DDA) mode, with data collected for the top 8 transitions [8]. For sample analysis, 2 μL of digested sample was injected, then eluted as follows: 0–12 min, B 7–40% at a 0.3 mL.min^−1^ flow rate; 12–13.5 min, B 90% at a 0.3 mL.min^−1^ flow rate; 13.5–16.5 min, B 95% at 0.4 mL.min^−1^ at a 0.4 μL.min^−1^ flow rate, 16.5–19 min, B 7% at 0.3 mL.min^−1^ flow rate. Data were collected using a 3 *m*/*z* isolation window, 3.5 kV spray voltage, 17 μA spray current, 10 aux gas flow rate, 45 sheath gas flow rate, 320 °C capillary temperature, normalised collision energy of 24, and a scan range of 100–5000 *m*/*z*.

Data were processed in Thermo Proteome Discoverer 2.1.0.81 (Thermo Fisher Scientific, Waltham, MA, USA) using SEQUEST HT, searching against the FASTA database GlutenALL_V1 [8]. The enzyme was ‘unspecified’, minimum peptide length 6, maximum peptide length 40, precursor mass tolerance 5 ppm, and fragment mass tolerance 0.025 Da. Results were filtered by an XCorr > 2. Label-free quantitation was undertaken by extraction of the precursor ion area. To calculate the total peptide family abundance, the area under the curve and error for each precursor ion were summed to produce a ‘total abundance’. Peptides were only included in the analysis if found within all biological replicates.

### 2.6. Enzyme-Linked Immunosorbent Assay

Enzyme-linked immunosorbent assay (ELISA) was undertaken using GlutenTox ELISA Competitive G12 (96-well) kits according to the manufacturer’s instructions (Biomedal Diagnostics, Spain). Briefly, in vitro digesta were freeze-dried (VirTis Genesis Pilot Lyophilizer), then protein extracted by dissolution and inversion in ‘extraction solution’ for 40 min at 50 °C. The resulting supernatant was collected by centrifugation at 10,000× *g* for 7 min, then diluted using ‘dilution solution’ to a gluten content between 50 and 300 ppm (within the central region of the standard curve). The diluted sample was then mixed with ‘GlutenTox G12-HRP conjugated antibody solution’ at 50:50, then incubated at 20 °C for 60 min. Aliquots of this solution (200 μL) were added to each well for 60 min, then washed five times with ‘wash solution’. ‘Substrate solution’ (100 μL) was added for 15 min, and then 100 μL of ‘stop solution’ quenched the reaction. The absorbance was read at 450 nm, and reactive gluten concentration was determined using second-order polynomial regression of the standard curve. Statistical analyses were undertaken in Prism. All samples were analysed in biological duplicate with experimental triplicates.

## 3. Results

Loaves of sourdough and fast fermentation bread were prepared as described in Section 2.2. Both protocols used the same raw ingredients and ratios. Notably, sourdough bread was prepared using an active type 1 sourdough culture as the leavening agent. In contrast, the fast fermentation control used high-speed mechanical dough development employing *S. cerevisiae* as the leavening agent. After baking, both loaves were freeze-dried to account for any differences in their water content. The following analysis examines the effect of sourdough fermentation on gluten proteins. Gliadin is the specific focus of this work due to its immunodominance in celiac disease [10].

### 3.1. HPLC

HPLC was undertaken to explore the degree of α/β-gliadin, γ-gliadin, and ω-gliadin protein proteolysis during bread preparation. Gliadin proteins were extracted from dough and bread using an ethanol extraction protocol (Section 2.4). A decrease in protein concentration using this protocol can occur through two mechanisms: (a) the proteolysis of gliadin proteins during fermentation or (b) the crosslinking of gliadin to the gluten macropolymer backbone preventing its extraction.

**Figure 1:**
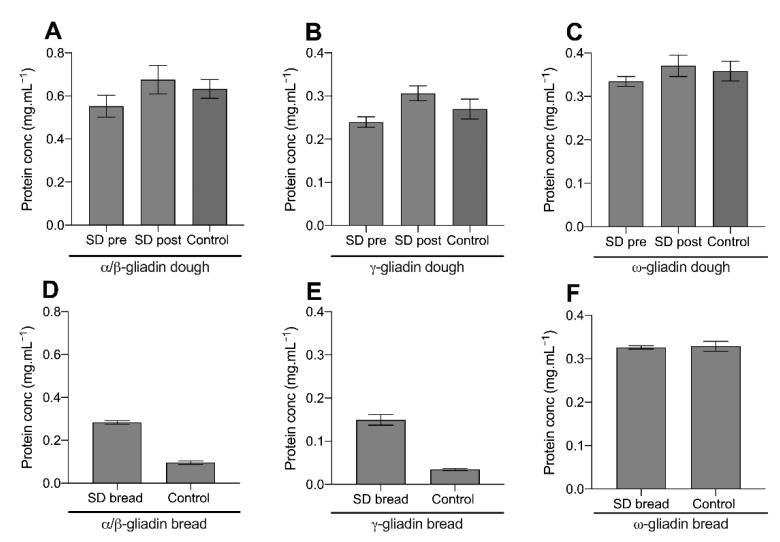
Extractable gliadin protein concentrations from sourdough and fast fermentation control products determined using HPLC. Gliadin proteins were extracted from sourdough before (pre) and after (post) dough fermentation and from the sourdough bread. Gliadin proteins were extracted from the fast fermentation control dough and bread. The concentration of extractible (**A**) α/β-gliadin from doughs, (**B**) γ-gliadin from doughs, (**C**) ω-gliadin from doughs, (**D**) α/β-gliadin from bread, (**E**) γ-gliadin from bread, and (**F**) ω-gliadin from bread. Samples were analysed in duplicate. Error bars display the standard deviation of the mean.

Figure 1 displays the concentration of extractable α/β-gliadin, γ-gliadin, and ω-gliadin proteins in the sourdough sample pre- and post-dough fermentation (upon dough formation and after overnight fermentation, respectively), the fast fermentation control dough, and both breads after baking. The post-fermentation sourdough sample and control dough are comparable equivalents. Figure 1A–C displays the extractable gliadin concentration from the doughs investigated. The concentrations of extractible α/β-gliadin (Figure 1A), γ-gliadin (Figure 1B), and ω-gliadin (Figure 1C) from the post-fermentation sourdough dough were not different (*p*-value > 0.05) when compared to the pre-fermentation sample and fast fermentation control. This suggested that sourdough fermentation did not proteolyse the gliadin proteins investigated.

In both sourdough and the control bread, baking significantly reduced the concentration of extractible α/β- and γ-gliadin, but not the ω-gliadin fraction (Figure 1D–F). Notably, this decrease in extractability was more pronounced in the control bread than the sourdough. Upon baking sourdough bread, the α/β-gliadin concentration decreased from a mean of 0.67 mg.mL^−1^ to 0.27 mg.mL^−1^ (mean decrease of 60%), while the γ-gliadin concentration decreased from 0.31 mg.mL^−1^ to 0.14 mg.mL^−1^ (mean decrease of 55%). Comparably, in the fast fermentation control, baking decreased the concentration α/β-gliadin from 0.63 mg.mL^−1^ to 0.09 mg.mL^−1^ (85% decrease) and the γ-gliadin from 0.27 mg.mL^−1^ to 0.03 mg.mL^−1^ (89% decrease). Baking decreased the concentration of extractible α/β- and γ-gliadin proteins in sourdough bread to a lesser extent than the fast fermentation control. 

Overall, HPLC analysis suggested that gliadin proteolysis was not occurring during fermentation with the sourdough starter culture investigated. However, sourdough fermentation altered the proportion of α/β- and γ-gliadin extraction after baking, suggesting that changes in gluten protein structure and/or the ability of gliadin to form thermally induced crosslinks.

### 3.2. Targeted Mass Spectrometry Analysis of Immunogenic Gluten Peptides

The concentration of six immunogenic gluten peptides deemed P1–P5 (see Section 2.5.2, Table 1) were monitored throughout static in vitro digestion [29] using targeted, quantitative label-free LC-MS (Section 2.5.2) [8,30]. Peptides P1–P5 displayed a burst-release profile; they were not detected during gastric digestion (0–120 min) but rapidly reached peak concentration in the intestinal phase (120–240 min), with all peaking between 130 and 140 min. Conversely, P6 was released gradually throughout the simulated in vitro digestion. Both observations are consistent with the previous release profiles from within a bread matrix [8]. P6 is compositionally different from P1–P5, derived from a different α-gliadin parent protein; however, the precise mechanism driving this difference in digestion is unknown. 

Once reaching peak concentration between 130 and 140 min of digestion, the concentration of P1–P5 within sourdough bread remained relatively stable and changed to a lesser extent than in the control bread. For example, the mean concentration of P2 at 140 min within sourdough bread was 2.29 μg.mL^−1^ (±0.46), then 2.51 μg.mL^−1^ (±0.06) at 240 min of digestion. In the fast fermentation control the mean concentration of P2 at 140 min was 3.85 μg.mL^−1^ (±0.36), then 2.59 μg.mL^−1^ (±0.22) at 240 min of digestion. For P1–P5, the decrease in peptide concentration after the 130–140-min peak to 240 min end point was statistically significant in the control fast fermentation bread (*p*-value of <0.05), but not in the sourdough sample (*p*-value > 0.05). This observation suggested that P1–P5 experienced further proteolysis during in vitro digestion in the control bread, but not the sourdough. 

**Figure 2:**
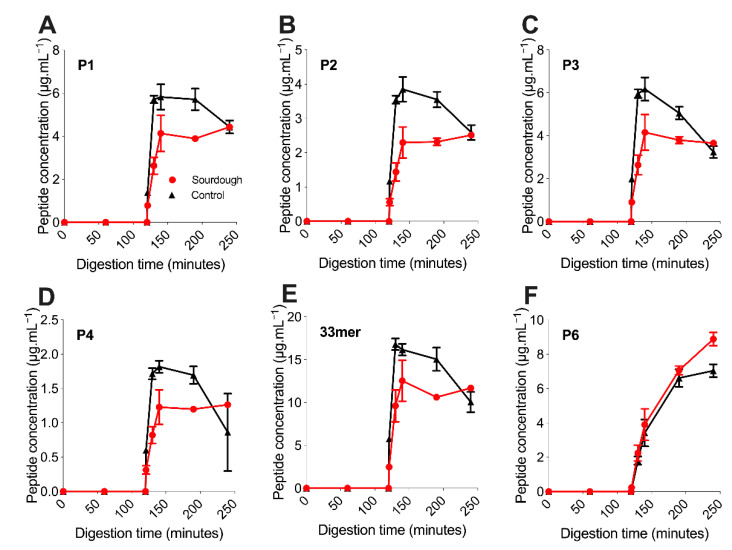
The release profiles of six key immunogenic gluten peptides throughout the in vitro digestion of sourdough (red) and fast fermentation control (black) breads. Peptide concentration determined by targeted LC-MS. (**A**) Peptide P1, (**B**) peptide P2, (**C**) peptide P3, (**D**) peptide P4, (**E**) peptide P5/33mer, and (**F**) peptide P6. All samples were digested and analysed in triplicate. Error bars display the standard deviation of the mean.

The concentration of P1–P5 throughout the digestion of sourdough bread was lower than the fast fermentation control with statistical significance (*p*-value < 0.05) (Figure 2A–E). This observation was in contrast to P6, whose concentration did not differ with statistical significance between the sourdough and control breads (*p*-value > 0.05). For example, at 130 min, the concentration of P1 in sourdough was 2.63 ± 0.39 μg.mL^−1^ versus 5.71 ± 0.17 μg.mL^−1^ in the control bread (mean decrease of 53 ± 7%). Similarly, the concentration of P4 in sourdough was 0.822 ± 0.124 μg.mL^−1^ versus 1.72 ± 0.08 μg.mL^−1^ in the control bread (mean decrease of 52 ± 8%). Similarly, the P2 concentration in sourdough decreased by a mean of 59 ± 8% at 130 min when compared to the control, P3 55 ± 8% and P5 42 ± 11%. This observation suggested that sourdough fermentation had decreased the concentration of immunogenic gluten peptides P1–P5, but not P6. 

### 3.3. Untargeted Mass Spectrometry Analysis of Gluten

Hundreds of peptides are produced during the digestion of gluten proteins [9,32]. Untargeted LC-MS was undertaken to detect these. The total number of gluten-derived peptides (non-immunogenic) varied throughout in vitro digestion. Figure S1 highlights the number of gluten-derived peptides identified in the digesta of both sourdough and the control bread, and both displayed equivalent global digestion profiles. More than 300 peptides were identified during the gastric phase of digestion for both bread types investigated. This number rapidly reduced to approximately 175 peptides on entry into the intestinal phase (45% decrease in peptides), an observation attributed to rapid intestinal protease digestion (trypsin and chymotrypsin) that is less constrained in terms of cleavage sites than gastric (pepsin) digestion. Over the remainder of intestinal digestion, the number of peptides gradually increased, suggesting peptides partially resistant to proteolytic digestion degraded into smaller peptide fragments. This hypothesis was supported by a decrease in the average peptide length throughout digestion (Figure S1). Overall, the number of peptides identified in the digesta of sourdough and the control bread did not differ with statistical significance and resembled those previously observed within the literature [8,9]. 

Immunogenic peptides were identified from peptide spectrum matches by the presence of nine amino acid epitopes [3]. Table 2 displays the total number of immunogenic gluten peptides detected at each digestion interval. The number of immunogenic peptides detected was significantly lower than the number of non-immunogenic peptides (Figure S1). Unlike the non-immunogenic peptides, which decreased by around 45% on entry into the intestinal phase, the number of immunogenic peptides did not significantly decrease. A slight increase in the number of immunogenic peptides occurred throughout intestinal digestion, increasing from 29 to 45 in sourdough digesta, and 30 to 36 for the control bread. Overall, there was a difference at some time points in the number of immunogenic gluten peptides identified throughout simulated digestion of the sourdough and control products. However, these differences did not exhibit an obvious trend. 

### 3.4. Analysis of P1–P6 Proteolytic Products

**Figure 3:**
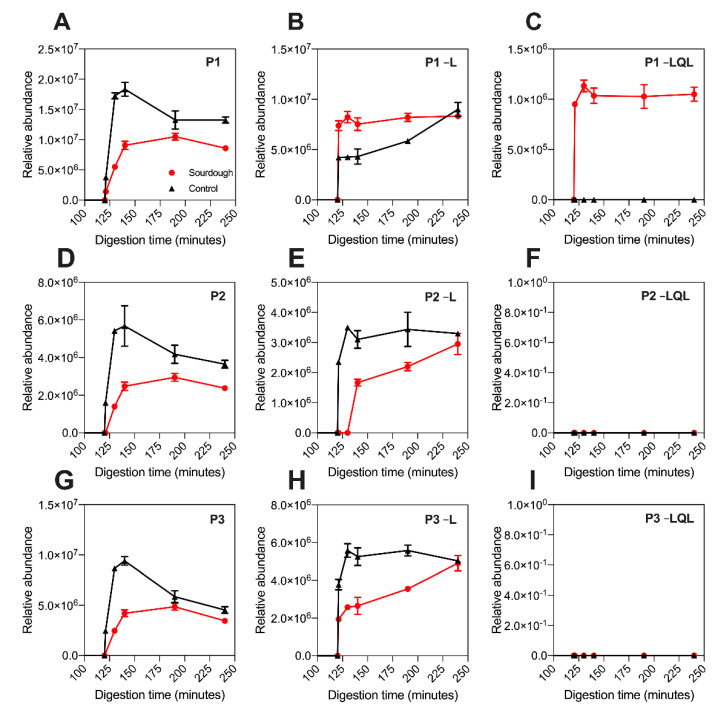
The release profile and relative abundance of the proteolytic products deriving from peptides P1, P2, and P3 throughout the digestion of sourdough (red) and the control (black) breads. Relative abundance determined by untargeted LC–MS. Release profile of (**A**) peptide P1, (**B**) P1-L proteolytic product, (**C**) P1-LQL proteolytic product, (**D**) peptide P2, (**E**) P2-L proteolytic product, (**F**) P2-LQL proteolytic product, (**G**) peptide P3, (**H**) P3-L proteolytic product, and (**I**) P3-LQL proteolytic product. L represents leucine and LQL represents a Leu-Gln-Leu tripeptide. All samples were digested and analysed in triplicate. Error bars display the standard deviation of the mean.

A proteolytic product is defined as a shorter peptide related to a peptide fragment by enzyme cleavage. Untargeted LC–MS was employed to investigate the presence of P1–P5’s proteolytic products. As displayed in Figure 3, the release profile of P1-P6 detected using untargeted LC–MS was equivalent to that observed using targeted LC–MS (Figure 2). Notably, the P4 peptide was not detected in sourdough digesta using untargeted LC–MS (in contrast to targeted LC–MS), which was attributed to its low concentration and the top N abundant precursor ion selection parameters applied during data collection (Section 2.5.3). 

Proteolytic products of P1, P2, P3, P4, and P5 were detected in the digesta of both sourdough and the control bread (Table 3). The detection of these peptides in both bread types suggested that they are the product of in vitro digestion. The proteolytic products detected were devoid of either a terminal leucine residue (referred to hereafter in three letter amino acid code) or terminal Leu-Gln-Leu tripeptide motif, and they were named accordingly, as highlighted in Table 3. Proteolytic products devoid of Leu were detected in the digesta of both sourdough and the control bread. In contrast, those devoid of the Leu-Gln-Leu tripeptide were only detected in sourdough digesta (Figure 3). 

The relative peptide abundance was determined by extracting the precursor ion area (Section 2.5.3). As highlighted in Figure 3 and Figure S2, the abundance of the proteolytic products devoid of leucine was approximately 50% higher in the digesta of sourdough bread at 130–140 min when compared to the control fast fermentation bread. For example, the abundance of P5–L at 140 min in sourdough was 8,800,000 units (±848,528), whereas in the control sample it was 4,200,000 units (±14,142). Notably, this increase in proteolytic product abundance in sourdough was proportional to the decrease in parent peptide concentration observed in Figure 2. This suggested that during digestion of sourdough bread, the parent peptides were being degraded into the –Leu proteolytic products more rapidly than the control bread. 

To determine if an overall decrease in the concentration of these peptide families had occurred during sourdough fermentation, the total abundance of the parent peptides plus proteolytic products was determined (Section 2.5.3). As highlighted in Table 4, the total abundance of peptides P1–P5 did not differ with statistical significance between the sourdough and control bread digesta (Table 4). This observation suggested that sourdough fermentation had not fully proteolysed any fraction of P1–P5, as initially suggested by the targeted LC–MS. 

Notably, the release profile of the proteolytic products differed between the sourdough and control breads. In sourdough digesta, the proteolytic products rapidly reached peak abundance within the intestinal phase of digestion. In contrast, their production in the control bread digesta was gradual, peaking at 240 min of in vitro digestion.

### 3.5. ELISA

**Figure 4:**
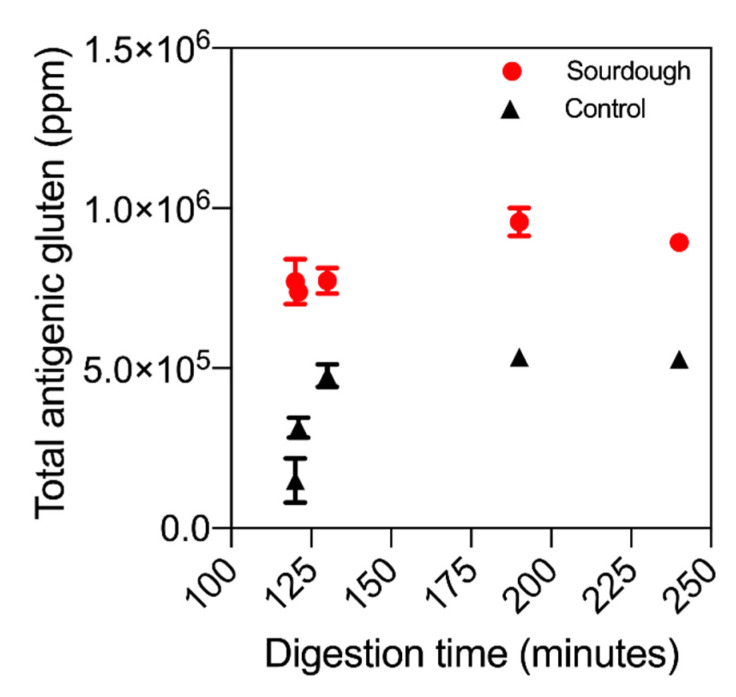
The reactive gluten throughout the simulated digestion of the sourdough (red) and control (black) bread products. Biological duplication and technical triplication were employed. Error bars display standard deviation of the mean.

ELISA analysis was used to investigate the total concentration of reactive gluten present throughout the in vitro digestion of sourdough and fast fermentation bread. Figure 4 displays the total antigenicity profile of both sourdough bread and the control throughout in vitro digestion. Perhaps surprisingly, the antigenicity of sourdough digesta was higher than that of the control bread. At 130 min, the reactive gluten concentration in sourdough digesta was 773,499 ppm (±140,306), while in the control it was 534,904 ppm (±29,007), amounting to a mean difference of 30%. This observation is partially attributed to differences in protein extractability due to sourdough fermentation, a known facet of the ELISA method. ELISA supported the untargeted LC–MS results that sourdough fermentation had not decreased the overall concentration of antigenic gluten. 

Additionally, the release profile of antigenic gluten in sourdough differed to the control bread. As illustrated in Figure 4, the concentration of antigenic gluten in sourdough digesta did not change throughout digestion (no change in statistical significance throughout, *p*-value > 0.05). Conversely, in the control bread the concentration followed an equivalent release profile to that observed for P1–P5, gradually increasing in antigenicity throughout digestion. 

## 4. Discussion

Throughout the literature, studies have demonstrated that sourdough starter cultures optimised for efficient gluten degradation under specific conditions can degrade the immunogenic peptide antigens that activate celiac disease [17,23]. In this study, we investigated the effect of one artisan sourdough culture on the immunogenic gluten fraction. This work differs from previous studies in two ways. Firstly, it uses a sourdough starter representative of those consumed by the public. Secondly, it uses a combination of targeted and discovery mass spectrometry plus immunoassays to monitor the effect of sourdough fermentation on gluten. Gliadin proteins and gliadin derived peptides were the focus of this study due to their immunodominance in celiac disease [10]. The key finding from this study was that sourdough fermentation, using the culture investigated herein, did not degrade immunogenic gluten proteins directly but altered their ability to be digested during in vitro simulated digestion. 

HPLC was initially undertaken to monitor the rate of gliadin degradation during sourdough fermentation (Section 3.1). HPLC demonstrated that gliadin was not degraded by the sourdough culture used herein. Interestingly, a difference in the extractability of α/β- and γ-gliadin proteins after baking was observed. When compared to the control, higher concentrations of these gliadins were extracted following the baking of sourdough bread. The α/β- and γ-gliadin proteins are often referred to as the ‘sulfur-rich’ gliadins, referencing the presence of cysteine residues within their primary sequence [13]. In contrast, the ω-gliadin fraction is referred to as ‘sulfur-poor’ due to the absence of cysteine residues. During baking, it is well known that the cysteine residues within the α/β- and γ-gliadin proteins undergo heat-induced polymerisation, forming disulfide crosslinks with the gluten macropolymer backbone and preventing their extraction from the food matrix [33,34]. Comparatively, the ω-gliadin fraction cannot form these crosslinks, explaining the absence of changes to extractability after baking [33]. The higher concentration of α/β- and γ-gliadin in sourdough bread suggests that fermentation altered the ability of these proteins to form disulfide bonds. Sourdough fermentation has been previously demonstrated to depolymerise the gluten macropolymer, significantly altering its structure [18,35]. Confocal microscopy (Figure S3) demonstrated that gluten depolymerisation had occurred within the sourdough bread, herein supporting that a change in protein structure had occurred. 

The sourdough and fast fermentation control breads were digested using a simulated human in vitro digestion protocol [29]. A simulated digestion was employed to gain a deeper understanding of the celiac antigens produced during human digestion. LC–MS was undertaken to investigate the identity and quantity of peptides produced during the digestion of sourdough bread versus the control. The focus of this work was six peptides (P1, P2, P3, P4, P5, and P6), selected due to their immunodominance in celiac disease [10,31]. The in vitro release profile of P6 differed from P1–P5, consistent with previous analyses [8,31]. P6 displayed a gradual release profile while P1–P5 a burst-release profile. This observation was attributed to differences in the parent protein’s digestibility and amino acid composition. Analysis of a gluten FASTA database [8] shows that the N-terminal residue in the P6 parent protein is phenylalanine (Phe), whereas in P1–P5, it is commonly tyrosine (Tyr). This difference likely altered the rate of bond cleavage; however, the mechanism is currently unknown. 

Initially, targeted LC–MS suggested that sourdough fermentation had decreased the concentration of five key immunogenic gluten peptides (P1–P5). Further analysis using untargeted LC–MS conversely demonstrated a proportional increase in the proteolytic products of these peptides. The proteolytic products detected were devoid of a leucine residue or Leu-Gln-Leu tripeptide. No epitopes are disrupted by the loss of these residues [36], leading to a hypothesis that the immunogenicity of the proteolytic products is likely unchanged. The ELISA analysis within Section 3.5 supports this hypothesis (discussed below). Based on these findings, we hypothesise that the sourdough products consumed by the public likely contain similar levels of immunogenic gluten proteins compared to fast-fermentation bread. Screening of other sourdough cultures will be required to confirm this hypothesis. 

The cleavage events required to produce the –Leu and –Leu-Gln-Leu proteolytic products both involve the proteolysis of Leu-Gln bonds. This cleavage is attributed to the intestinal protease chymotrypsin [8]. Chymotrypsin is the most likely candidate for this cleavage event for three reasons. Firstly, the cleavage takes place in both the control and sourdough bread digesta, suggesting it is a consequence of the in vitro digestion. Secondly, the proteolytic products are produced only in the intestinal phase. Thirdly, in silico modelling (using ExPASy PeptideCutter [37]) of chymotrypsin proteolysis indicates that this specific cleavage event has a high likelihood of occurring.

The production of the –Leu and –Leu-Gln-Leu products could theoretically occur through three scenarios (Figure S4): (1) the ‘primary cleavage’ of the terminal Leu residue to produce –Leu products; (2) subsequent ‘secondary cleavage’ of the Gln-Leu dipeptide would produce the –Leu-Gln-Leu product, or alternatively, (3) the tertiary cleavage of the full tripeptide –Leu-Gln-Leu by cleavage between Leu-Gln-Leu and Gln-Pro bonds. Because the –Leu product was detected at higher concentrations than the –Leu-Gln-Leu product, we hypothesised that the primary cleavage was occurring faster than the secondary and tertiary cleavage events. This likely occurred because proline residues partially inhibit chymotrypsin. In the secondary cleavage event, a proline residue is required in the P2’ pocket [38], decreasing the likelihood of the reaction and concentration of product produced.

The digestion kinetics of P1–P5 and their proteolytic products varied between the sourdough and control breads. Several mechanisms may have contributed to this change. Sourdough fermentation partially proteolyses the gluten protein fraction [18,39,40]. Proteolysis alters the cleavage events required to produce peptides (such as those investigated herein), influencing the rate of digestion. Fermentation alters the degree of protein hydrolysis and the hydrolytic state of other constituents within the food matrix [35,40]. Changes to the structure of a food’s matrix can alter the pattern of protein digestion of gluten [41] and other food systems [42,43]. We observed changes in the structure of the gluten proteins in sourdough bread versus the control, supporting this hypothesis. Thirdly, HPLC demonstrates that sourdough fermentation increases the extractability/solubility of the α/β- and γ-gliadin proteins. Changes in solubility can significantly alter protein digestion by increasing the probability of substrate enzyme collisions. 

The observations and discussion throughout this manuscript surmise that the sourdough culture investigated herein did not directly proteolyse P1, P2, P3, P4, P5, and P6, but altered how they were digested in vitro. The mechanism driving this altered digestion profile is unknown but can be attributed to partial proteolysis that occurred during sourdough fermentation, which consequently caused changes to protein and food matrix structure. This work contains the most detailed analysis, to date, of gluten protein digestion after sourdough fermentation and highlights significant gaps in our knowledge surrounding the effect of sourdough fermentation on the identity and quantity of antigens involved in celiac disease. Further research is warranted to assess the effect of consumer-relevant sourdough cultures on antigenic gluten and allow the engineering of cultures that produce both altered antigenic gluten profiles and bread with an adequate sensory profile. 

## Figures and Tables

**Table 1:** The peptides investigated by targeted LC-MS. Amino acid sequence displays one letter amino acid code.

Peptide	Amino Acid Sequence
P1	LQLQPFPQPQLPY
P2	LQLQPFPQPQLPYPQPQPF
P3	LQLQPFPQPQLPYPQPHLPYPQPQPF
P4	LQLQPFPQPQLPYPQPQLPYPQPQPF
P5	LQLQPFPQPQLPYPQPQLPYPQPQLPYPQPQPF
P6	RPQQPYPQPQPQY
P1H	LQLQPF * PQPQLPY

* P1H, residue six was L-phenylalanine-^13^C9, ^15^N.

**Table 2:** The number of immunogenic peptides detected during in vitro digestion of sourdough and fast fermentation breads.

Digestion Time (Minutes)	60	120	121	130	140	190	240
Sourdough	21	33	29	41	40	41	45
Control	30	37	34	42	39	41	36

**Table 3:** The proteolytic products of peptides P1–P5 detected during the digestion of sourdough and fast fermentation bread. Amino acid sequence displays one letter amino acid code. L represents leucine and LQL represents a Leu-Gln-Leu tripeptide.

Name	Amino Acid Sequence
P1-L	QLQPFPQPQLPY
P1-LQL	QPFPQPQLPY
P2-L	QLQPFPQPQLPYPQPQPF
P3-L	QLQPFPQPQLPYPQPHLPYPQPQPF
P4-L	QLQPFPQPQLPYPQPQLPYPQPQPF
P5-L	QLQPFPQPQLPYPQPQLPYPQPQLPYPQPQPF
P5-LQL	QPFPQPQLPYPQPQLPYPQPQLPYPQPQPF

**Table 4:** The total relative abundance of peptides P1–P5 and their proteolytic products. Brackets display the summed standard error.

Total Peptide Abundance (Units)	Sourdough		Control	
P1	4.09 × 10^9^	(8.80 × 10^7^)	4.35 × 10^9^	(2.22 × 10^7^)
P2	7.63 × 10^8^	(3.42 × 10^7^)	6.86 × 10^8^	(2.34 × 10^7^)
P3	1.11 × 10^9^	(2.23 × 10^7^)	1.17 × 10^8^	(4.81 × 10^7^)
P4	5.14 ×10^8^	(1.72 × 10^7^)	5.50 × 10^8^	(1.33 × 10^7^)
P5	3.20 × 10^9^	(7.57 × 10^7^)	3.53 × 10^8^	(9.05 × 10^7^)

## Supplementary Materials

The following are available online at https://www.mdpi.com/article/10.3390/nu13061906/s1, File S1: Quantifier fragment ions, Figure S1: Non-immunogenic peptide trends and length, Figure S2: P4, P5 and P6 release profile, Figure S3: Confocal microscopy, Figure S4: The cleavage events required to produce the proteolytic products.
