# Supplementary material for: A Case Study of the Response of Immunogenic Gluten Peptides to Sourdough Proteolysis
Source: Nutrients. 2021 Jun 1;13(6):1906. doi: 10.3390/nu13061906 (PMC8229354; doi:10.3390/nu13061906)

Supplementary file 1. Quantifier Fragment ions

Quantifier fragment ions used during targeted mass spectrometry to identify the seven key immunogenic gluten peptides.

Peptide P1: precursor ion, 2+, 784.927 *m/z*; y2, 279.134; b4, 483.293; b6, 470.240; and b11 1290.719 *m/z.*

Peptide P1H: precursor ion, 2+, 787.927 *m/z*; y2, 279.134; b4, 483.293; b6, 476.254; b11, 1296.731 *m/z*.

Peptide P2: precursor ion, 3+, 755.068 *m/z*; y4, 488.251; b8, 952.526; b9, 1049.544 *m/z*.

Peptide P3: precursor ion, 3+, 1029.543 *m/z*; y2, 263.139; b7, 824.429; b6, 713.357; b8, 952.527; b11, 1290.722 *m/z*.

Peptide P4: precursor ion, 3+, 1032.543 *m/z*; y2, 263.139; y4, 488.250; y6, 713.358; y8, 973.479 *m/z*.

Peptide P5: precursor ion, 4+, 978.264 *m/z*; y4, 488.252; b21, 824.429; y8, 973.480; b11, 1290.726 *m/z*.

Peptide P6: precursor ion, 2+, 813.905 *m/z*; y3, 407.194; a8, 967.513; b9, 995.508 *m/z*.

Figure Supplementary 1. Non-immunogenic peptide trends and length

A) The number of non-immunogenic gluten derived peptides detected throughout *in vitro* digestion, error bars display the standard deviation of the mean, B) box and whisker graph displaying the decrease in peptide length in sourdough product, and C) box and whisker graph displaying the decrease in peptide length in the control product. Whiskers display the 5-95 percentile. Samples were analysed in biological triplicate and technical duplicate.

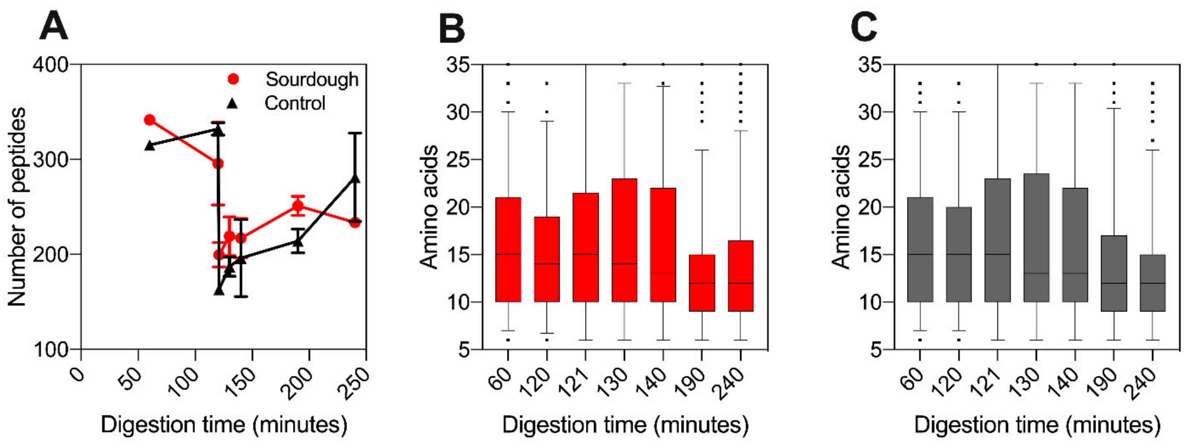

Figure Supplementary 2. P4, P5 and P6 release profile

The release profile and relative abundance of the proteolytic products deriving from P4, P5 and P6 throughout the digestion of sourdough (red) and the control (black) breads. Relative abundance determined by untargeted LC-MS. Release profile of A) peptide P4, B) P4–L proteolytic product, C) P4–LQL proteolytic product, D) peptide P5, E) P5–L proteolytic product, F) P5–LQL proteolytic product, and G) peptide P6. All samples were digested and analysed in triplicate. Error bars display the standard deviation of the mean.

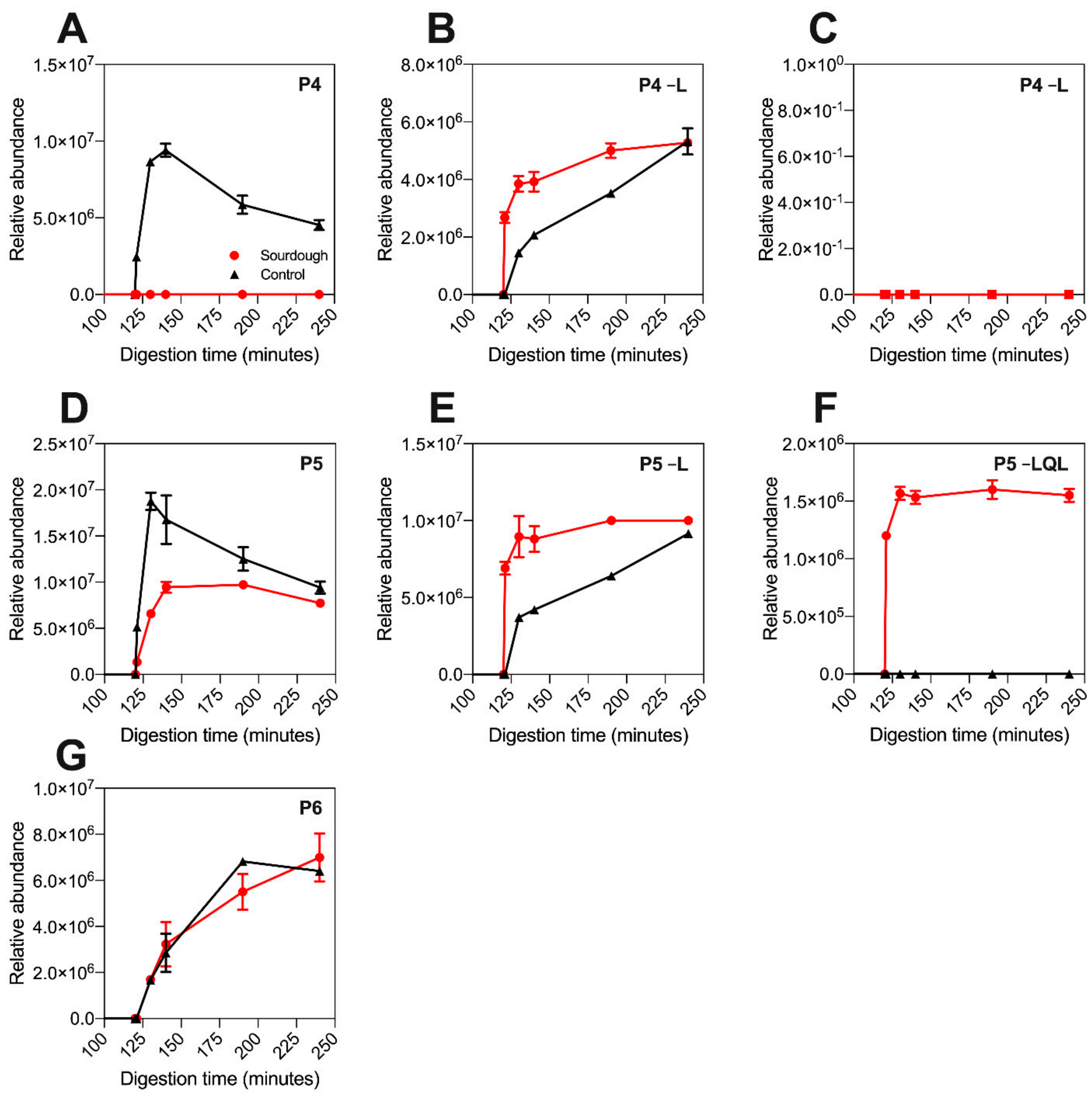

Figure Supplementary 3. Confocal microscopy

Confocal microscopy images representative of those taken of the sourdough and the control products highlighting the significant change in gluten structure within sourdough bread. A) gluten protein structure in sourdough product, B) gluten protein structure in control product. The protein network is shown in red (stained with Rhodamine B). Starch granules were stained with FITC (channel not displayed).

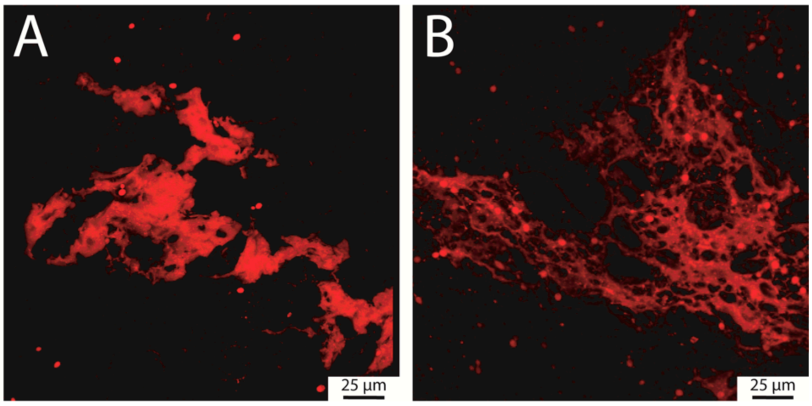

Figure Supplementary 4. The cleavage events required to produce the proteolytic products

The cleavage events required to produce the –L and –LQL proteolytic products of P1-P5, using the 33mer/P5 peptide as an example.

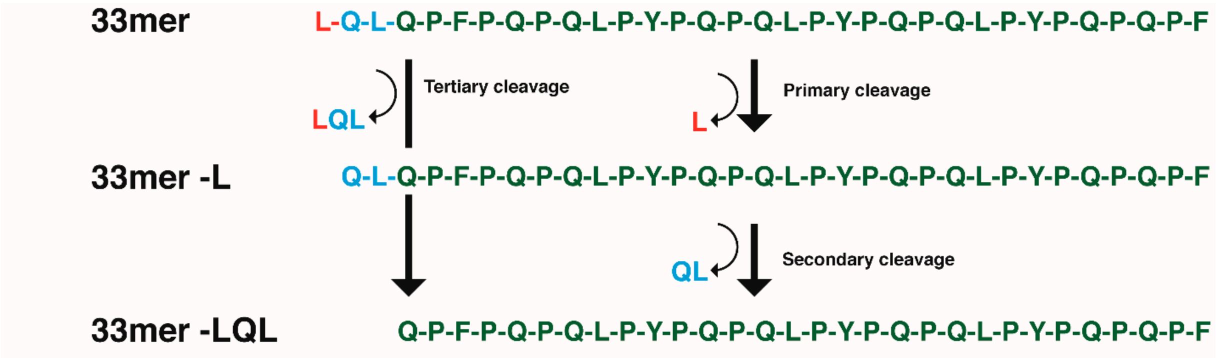

Supplement: Supplementary file 1 [file nutrients-13-01906-s001.zip › nutrients-1223667-supplementary.pdf]
